# Monthly biological larviciding associated with a tenfold decrease in larval density in fish farming ponds and reduced community-wide malaria incidence in northwestern Brazil

**DOI:** 10.1186/s13071-021-04964-3

**Published:** 2021-09-03

**Authors:** Pablo S. Fontoura, Marcos F. Silva, Anderson S. da Costa, Francismar S. Ribeiro, Marcílio S. Ferreira, Simone Ladeia-Andrade, Juliana Tonini, Priscila T. Rodrigues, Marcia C. Castro, Marcelo U. Ferreira

**Affiliations:** 1 Department of Parasitology, Institute of Biomedical Sciences, University of São Paulo, Av. Prof. Lineu Prestes 1374, São Paulo, SP 05508-900 Brazil; 2 Multidisciplinary Center, Federal University of Acre, Cruzeiro do Sul, Brazil; 3 Laboratory of Parasitic Diseases, Oswaldo Cruz Institute, Fiocruz, Rio de Janeiro, RJ 21045-900 Brazil; 4 Department of Global Health and Population, Harvard T. H. Chan School of Public Health, Boston, MA 02115 USA

**Keywords:** Malaria, *Anopheles*, Biological larvicides, Fish farming, Amazon

## Abstract

**Background:**

Larvicides are typically applied to fixed and findable mosquito breeding sites, such as fish farming ponds used in commercial aquaculture, to kill immature forms and thereby reduce the size of adult malaria vector populations. However, there is little evidence suggesting that larviciding may suppress community-wide malaria transmission outside Africa. Here, we tested whether the biological larvicide VectoMax FG applied at monthly intervals to fish farming ponds can reduce malaria incidence in Amazonian Brazil.

**Methods:**

This study was carried out in Vila Assis Brasil (VAB; population 1700), a peri-urban malaria hotspot in northwestern Brazil with a baseline annual parasite incidence of 553 malaria cases per 1000 inhabitants. The intervention consisted of monthly treatments with 20 kg/ha of VectoMax FG of all water-filled fish ponds in VAB (*n* ranging between 167 and 170) with a surface area between 20 and 8000 m^2^, using knapsack power mistblowers. We used single-group interrupted time-series analysis to compare monthly larval density measurements in fish ponds during a 14-month pre-intervention period (September 2017–October 2018), with measurements made during November 2018–October 2019 and shortly after the 12-month intervention (November 2019). We used interrupted time-series analysis with a comparison group to contrast the malaria incidence trends in VAB and nearby nonintervention localities before and during the intervention.

**Results:**

Average larval densities decreased tenfold in treated fish farming ponds, from 0.467 (95% confidence interval [CI], 0.444–0.490) anopheline larvae per dip pre-intervention (September 2017–October 2018) to 0.046 (95% CI, 0.041–0.051) larvae per dip during (November 2018–October 2019) and shortly after the intervention (November 2019). Average malaria incidence rates decreased by 0.08 (95% CI, 0.04–0.11) cases per 100 person-months (*P* < 0.0001) during the intervention in VAB and remained nearly unchanged in comparison localities. We estimate that the intervention averted 24.5 (95% CI, 6.2–42.8) malaria cases in VAB between January and December 2019.

**Conclusions:**

Regular larviciding is associated with a dramatic decrease in larval density and a modest but significant decrease in community-wide malaria incidence. Larviciding may provide a valuable complementary vector control strategy in commercial aquaculture settings across the Amazon.

**Graphical abstract:**

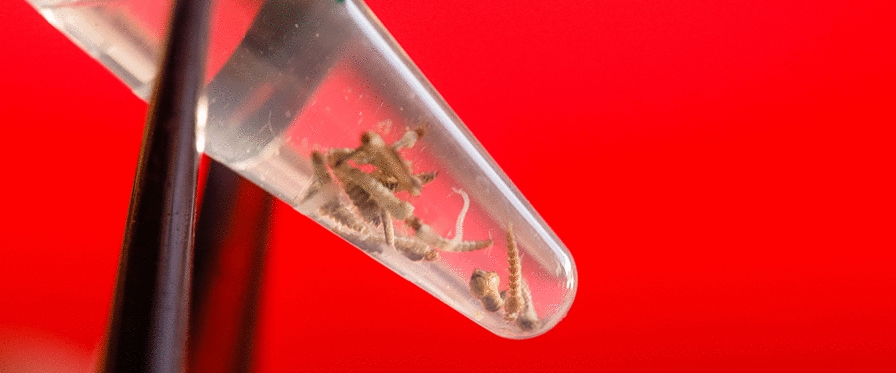

## Background

Despite significant progress towards malaria elimination over the past two decades, focal transmission persists in the Americas, where 889,000 cases were estimated to occur in 2019 [1]. The Amazon Basin, extending over nine countries of South America, contributes nearly 90% of the malaria burden on the continent [2].

Malaria transmission in the Amazon is greatest in farming settlements, mining camps, and riverine villages, and typically low in cities and towns [2, 3]. However, infections can also be acquired within and near urban centers across the region [4–9], where large populations of the primary local vector, *Anopheles darlingi*, thrive in natural and artificial water habitats. Human-made tanks or natural water bodies used for commercial aquaculture are increasingly common larval habitats in urban and peri-urban areas in the Amazon [7, 10–13].

The main malaria transmission pocket in the Amazon Basin of Brazil is the upper Juruá Valley, next to the border with Peru [14]. With < 0.5% of Amazon’s population, the region contributes ~ 8% of the overall country’s malaria burden, estimated at 157,454 cases in 2019 [1]. Malaria transmission in the Juruá Valley extends into urban areas, where large outbreaks have been associated with larval habitats next to human residences, which were originally opened or modified for fish farming [7, 11–13].

Larval source management, and particularly the application of biological larvicides**,** may supplement core interventions such as long-lasting insecticidal net (LLIN) distribution and indoor residual spraying (IRS) with insecticides. Larviciding is well suited to control outdoor feeding (exophagic) and outdoor resting (exophilic) mosquito vectors in densely populated areas with well-delineated, easy to find, and readily accessible breeding sites [15]. Fish farming ponds in urbanized spaces of the Juruá Valley offer a perfect fit for these criteria. First, the biting and resting behavior of *An. darlingi* has changed over the past decades [16], and this vector now feeds and rests predominantly outdoors (e.g., [17]). Second, it displays more intense human biting activity at dusk and a minor peak at dawn (e.g., [18]). Importantly, LLIN use is unlikely to prevent early-evening biting, except for infants who are more likely to sleep under a bednet at daytime, and IRS has little effect against exophilic and exophagic mosquitoes. Third, fish farming ponds in the region are fixed and findable water bodies that typically remain water-filled during the 5-month dry season. Nevertheless, it remains undetermined whether larviciding can reduce focal malaria transmission in commercial aquaculture settings next to cities and towns across the Amazon.

To address this critical knowledge gap, we assess the impact on malaria prevalence and incidence of community-wide larviciding of fish farming ponds in a peri-urban transmission hotspot in Brazil. We use an environmentally safe biological larvicide with robust residual activity (90–100% reduction in larval density) lasting for 35 days after retreatment of water habitats [19] and negligible effects on non-target populations, such as other invertebrates, fish, and humans. We tested whether larviciding at monthly intervals might reduce larval density in fish farming ponds leading to decreased malaria incidence in the intervention community, compared with untreated communities in the same region.

## Methods

### Study area

**Fig. 1:**
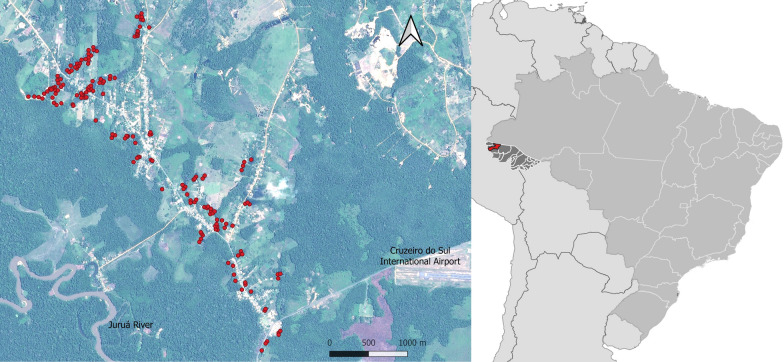
Study site (Vila Assis Brasil; VAB) in northwestern Brazil and the location of individual fish farming ponds that were treated with biological larvicides (red dots). The inset shows a map of Brazil, with Acre State highlighted in dark gray and the municipality of Cruzeiro do Sul (where VAB is situated) in red

The peri-urban village of Vila Assis Brasil (VAB; 07°35′30ʺS, 72°48′29ʺW), with ~ 1700 permanent residents, is part of the municipality of Cruzeiro do Sul (CZS; population 87,673), upper Juruá Valley, Acre State (Fig. 1). VAB is connected to the municipal seat of CZS, a city with ~ 63,800 inhabitants, by a 17-km paved road. Average monthly rainfall estimates from the Climate Hazards Group Infrared Precipitation with Stations (CHIRPS) data set, which uses modeled satellite-based infrared data (http://chg.geog.ucsb.edu/data/chirps), ranged, during the study period, from 241 to 332 mm in the rainy season (October–April) and from 52 to 138 mm in the dry season (May–September).

VAB experiences year-round malaria transmission, with an average annual parasite incidence of 553 cases per 1000 inhabitants between 2016 and 2018. *Anopheles darlingi* is the primary vector, but larvae of *Anopheles albitarsis* sensu lato (Galvão and Damasceno) are also abundant in nearby water bodies [12]. A relatively small proportion of the VAB population was covered by core vector control measures at the study baseline; only 58.6% of study participants reportedly slept under an LLIN in the preceding week, and 38.1% of participants’ houses had been sprayed with pyrethroid insecticides within the past 6 months.

### Monthly larval density monitoring pre-intervention

Fieldwork was carried out between September 2017 and November 2019. Over a period of 14 months prior to intervention (September 2017 through October 2018), we identified and georeferenced all fish farming ponds in VAB (Fig. 1). The average surface area of aquatic habitats was 1,208.86 m^2^ (ranging from 20 to 8000 m^2^). No other natural or artificial water bodies were characterized as major larval habitats (on the basis of both size and larval density) in the area. During that pre-intervention period, we measured the larval density in fish ponds monthly using a standard dipping technique for sampling water bodies [20]; 2–3 dips were taken, using a 500-ml ladle, every 3 m along the edges of the fish pond (total, 20–80 dips per water body). Larvae were classified as first (L1), second (L2), third (L3), or fourth (L4) instar, and then reclassified as early (L1 and L2) or late (L3 and L4) instars. The presence and number of pupae were recorded but not used in the analysis due to the impracticability of morphological differentiation of genera under field conditions. Larval density in each water body was calculated as the average count per dip. At the study outset, in September 2017, 11 of the 144 local fish ponds were empty. Of the 133 water-filled ponds examined, 112 (84.2%) were positive for immature anopheline stages; 131 (98.5%) of them supported active fish farming. Culicine larvae or pupae were found in 107 (80.5%) water-filled ponds at baseline. Additional ponds were excavated during the study period, while others were transiently emptied for fish harvesting or were abandoned. The number of ponds examined for larval density prior to intervention ranged from 127 in March 2018 (10 ponds were empty at the time of the survey) to 165 in October 2018 (four were empty).

### Biological larvicide application and post-treatment larval density monitoring

Starting in November 2018 and continuing through October 2019, we carried out monthly larviciding in all fish farming ponds of VAB. The biological larvicide used in this study was VectoMax FG, a granular formulation that combines toxins from *Bacillus thuringiensis* serovar *israelensis* (strain AM65-52) and *Lysinibacillus* (formerly *Bacillus*) *sphaericus* 2362 (strain ABTS-1743) in a single microparticle, with a potency of 50 international toxin units. These toxins, once ingested by larvae, lead to lysis of the insect’s gut epithelium. VectoMax FG (Valent BioSciences, Libertyville, IL, USA) at the concentration of 20 kg/ha was applied using 18-l capacity knapsack power mistblowers (Guarany, Itu, Brazil) operating at a walking speed of 0.5 m/s, with a reach of 10 m, covering a surface area of 5 m^2^/s [19].

Based on the extended residual effect of VectoMax FG applied to fish ponds described in the region [19], habitats were retreated monthly; the only exception was May 2019, when treatment failed due to an unexpected delay in the shipping of larvicides to the field site. As a consequence, there was a 2-month interval between the treatment applied in April 2019 and the next treatment in June 2019. Larval density in each habitat was monitored monthly (including May 2019), 21 days after (re)treatment, exactly as done before the intervention. Note that the last larval density measurement was made in November 2019, exactly 21 days after the last larvicide treatment (in mid-October 2019). The number of water-filled ponds that were treated with biological larvicides and monitored for larval density ranged from 167 to 170 between November 2018 and October 2019. No habitat was treated in November 2019, but all fish ponds were monitored for larval density. VAB is located > 10 km from other villages with similar malaria epidemiology that served as comparison localities, thus preventing spill-over effects on anopheline populations.

### Cross-sectional malaria prevalence surveys

Malaria prevalence rates in VAB were measured before and during the intervention. To this end, we surveyed the entire population of VAB in August–September 2018 (prior to larvicide treatment) and in March 2019 and September–October 2019 (during larvicide treatment). During each survey, we collected a finger-prick capillary blood sample from consenting individuals aged ≥ 3 months for malaria diagnosis by microscopy and real-time PCR. At least three visits were made before a house was considered transiently or permanently uninhabited. Because of temporary absences and refusals, the number of study participants screened for malaria parasites during each survey varied between 1185 and 1500 (microscopy) and between 1076 and 1498 (real-time PCR).

For on-site microscopic diagnosis of malaria, thick blood smears were stained with Giemsa and read by a local expert microscopist within 24 h. At least 200 thick smear fields were examined at × 1000 magnification before a slide was declared negative. Infections diagnosed by microscopy on-site were promptly treated as per current malaria treatment guidelines in Brazil [21]. For confirmatory molecular diagnosis, 50-μl aliquots of finger-prick blood samples were used to isolate parasite DNA, using the QIAsymphony investigator kit (Qiagen, Hilden, Germany). Participants with available 50-μl blood aliquots for DNA extraction were screened for malaria parasites using a highly sensitive real-time PCR protocol that targets a genus-specific segment of the mitochondrial *cytochrome b* (*cytb*) gene [22], with a detection threshold of ~ 0.5 parasites per μl of blood. Oligonucleotide primer sequences were previously described [22]. Amplifications were run on a QuantStudio 6 Flex real-time PCR system (Applied Biosystems, Foster City, CA, USA) with the following cycling program: 95 °C for 10 min, followed by 40 cycles of 15 s at 95 °C and 1 min at 60 °C. No-template negative controls, containing all reagents for amplification except for the DNA template, were run for every PCR microplate.

### Malaria surveillance data

We retrieved all notifications of malaria cases diagnosed between September 2017 and December 2019 in CZS that were entered into the electronic malaria notification system of the Ministry of Health of Brazil. Because malaria is a notifiable disease in Brazil and diagnostic testing and treatment are not available outside the network of government-run healthcare facilities, the case notification database is estimated to comprise 99.6% of all laboratory-diagnosed malaria cases countrywide [23]. These data combined with population size estimates obtained during periodic census surveys carried out by the local malaria control program were used to calculate the monthly incidence of malaria in VAB and the contrast group (see below) before and during the larviciding intervention.

For operational malaria control purposes, CZS is divided into smaller geographic units, or “localities,” with shared ecoepidemiological characteristics [24]. Seven localities in CZS were chosen for inclusion in the nonintervention contrast group using the criteria and approach described under Data analysis.

### Data analysis

We used a quasi-experimental design to compare larval density and malaria incidence rates before and during the intervention in VAB while controlling for confounding from secular trends and other contextual changes over time [25]. We define intervention as the repeated application of larvicides, at monthly intervals, from November 2018 through October 2019. We used single-group interrupted time-series analysis (ITSA), with the variables *T* (time since the start of the study), *X* (dummy variable representing the intervention), and *XT* (an interaction term), to measure the change in average larval density after introducing the intervention. The first regression line was fitted to both pre- and post-intervention larval density data, with the slope corresponding to the secular trend, while a second line was fitted to capture the deviation of the post-intervention data from the first line (change in level and/or trend). Calculations were made with the *itsa* command in STATA version 14.1 (Stata, College Station, TX, USA), which uses an ordinary least-squares regression model with Newey–West standard errors to adjust for autocorrelation of residuals over time (events closer together in a time series tend to be more similar than events further apart in time) [26]. This analysis was also adjusted for rainfall 15 days prior to larval sampling, which was positively correlated with larval density measured before and during the intervention (*r* = 0.799, *P* < 0.001). To this end, we added an independent variable (precipitation in mm) to the standard ITSA procedure as described [26]. Statistical significance was defined at the 5% level (two-tailed tests) and 95% confidence intervals (CI) were estimated whenever appropriate.

We also used ITSA with a comparison group to test whether the decrease in malaria incidence was greater in VAB than in nonintervention localities starting in January 2019. We defined a 2-month lag period before any effect of larviciding on malaria incidence rates in VAB was expected (November–December 2018) because: (a) infectious adult mosquitoes that were already present in the area would not be affected by larviciding and might continue transmitting malaria, and (b) following infectious mosquito bites, individuals take at least 2 weeks to develop malaria symptoms and seek treatment. ITSA with a nonintervention group, compared with single-group ITSA, included four additional variables: *Z* (dummy variable to denote the treatment assignment, whether intervention or control) and the interaction terms *XT*, *ZT*, and *ZXT*. Comparison localities in CZS had to meet three selection criteria: (i) no significant difference in malaria incidence rate, compared with VAB, prior to intervention, (ii) no significant difference in the trend of malaria incidence over time, compared with VAB, prior to the intervention, with significance here defined at the 10% level [26], and (iii) distance > 10 km from VAB to avoid spill-over effects of the intervention. Seven rural localities, with a combined population of 3,740 residents, were chosen to form a comparator group: Boca do Moa, Igarapé Preto, São José, Santa Bárbara, Santa Rosa, Santa Luzia (BR-364), and Boca da Alemanha. Because no microscopy- or PCR-based malaria prevalence data were available for localities other than VAB, no attempt was made to evaluate the impact of larviciding on malaria prevalence using ITSA with a nonintervention group.

## Results

### Reduced density of anopheline larvae in fish ponds during larvicide application

**Fig. 2:**
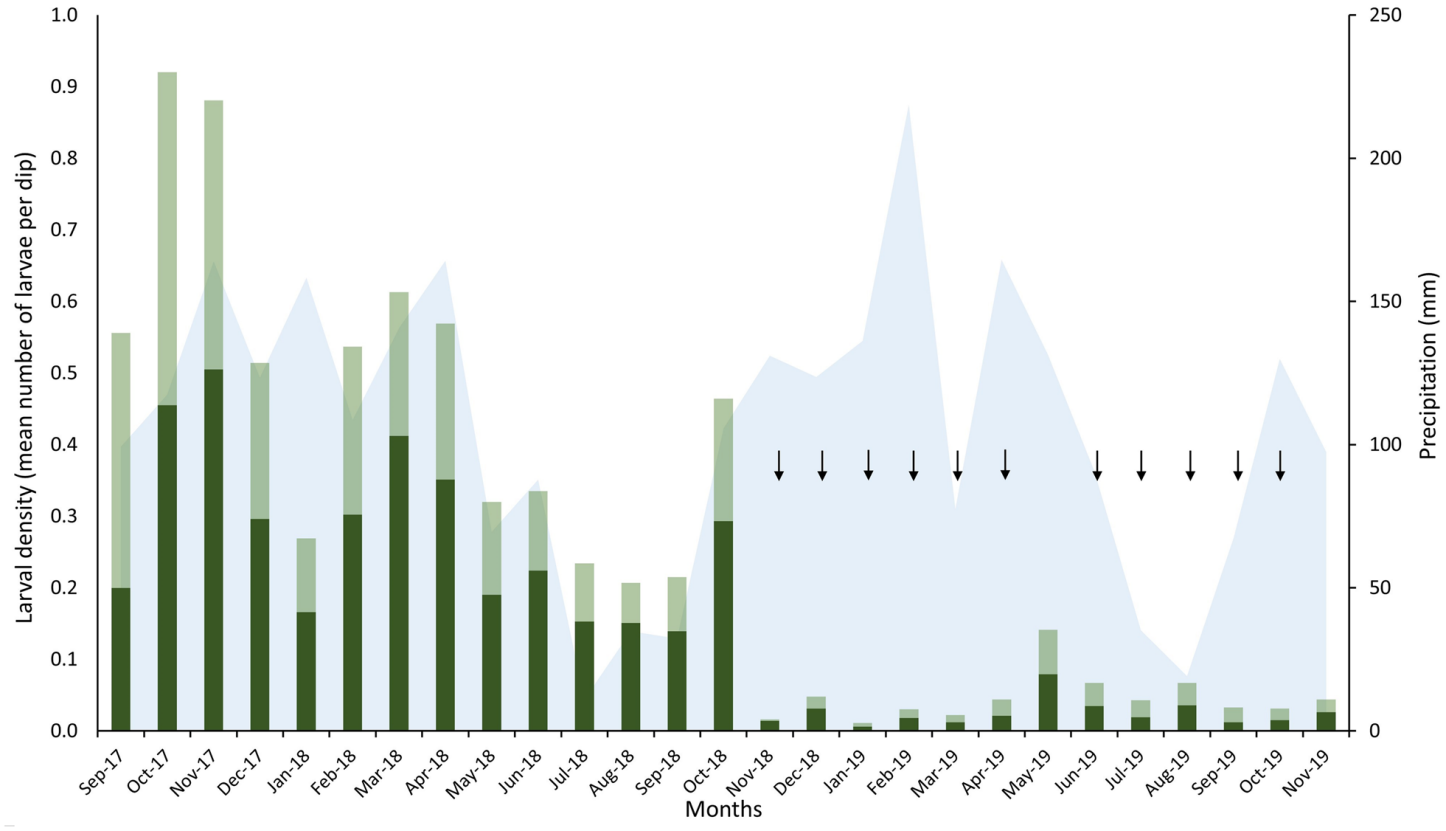
Monitoring of anopheline larval density in fish farming ponds of Vila Assis Brasil (VAB), northwestern Brazil, from September 2017 through November 2019. Monthly averages of larvae per dip are shown for early instar (L1 and L2; dark green) and late instar larvae (L3 and L4; light green) collected in fish farming ponds in VAB (number of ponds ranging between 127 and 165 before the intervention and between 167 and 170 during and shortly after the intervention). Vertical arrows indicate the timing of larvicide treatment (from October 2018 through April 2019 and from June 2019 through October 2019). Larval density measurements were carried out 21 days after each larvicide application during the intervention. The light blue area chart indicates the monthly rainfall (mm)

**Fig. 3:**
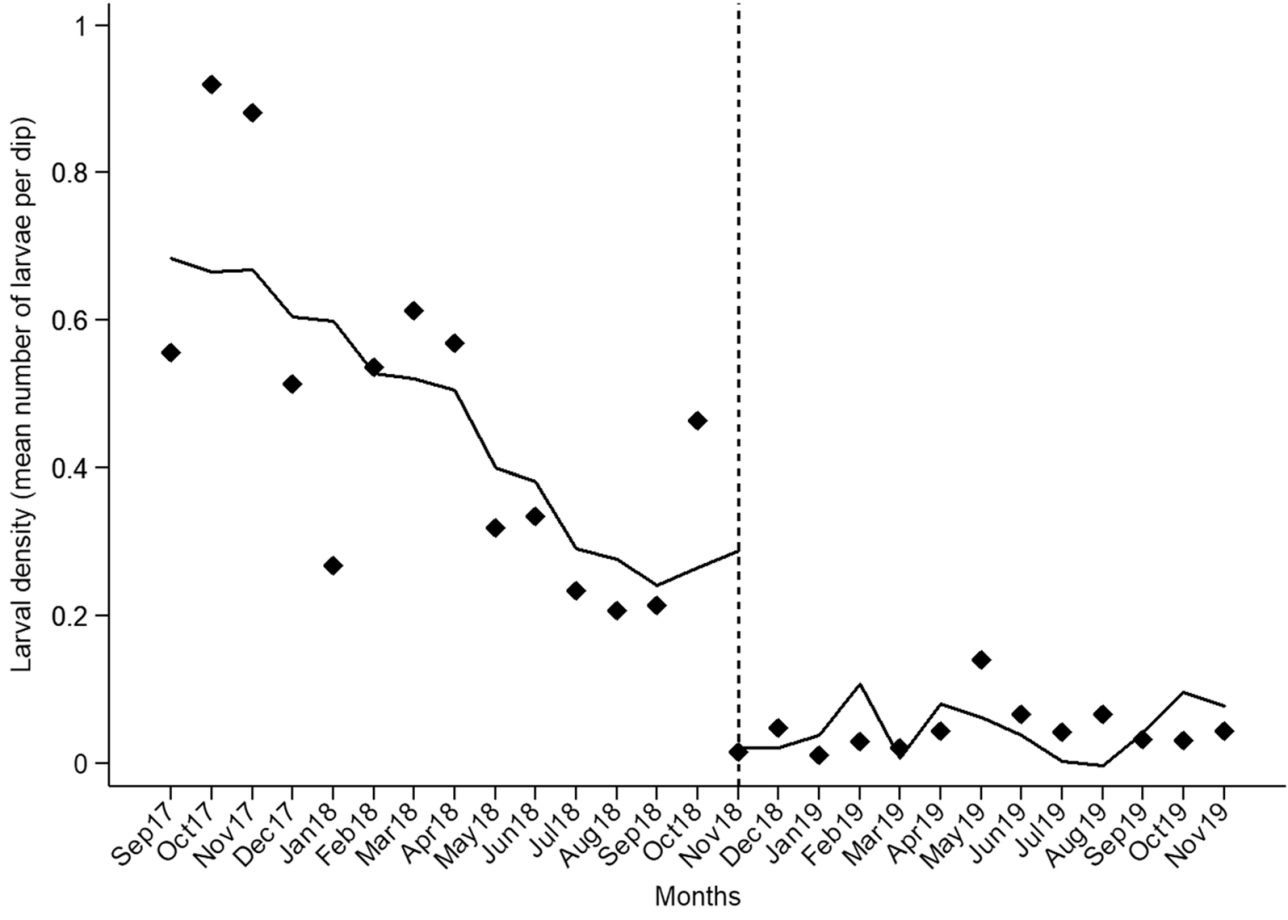
Interrupted time-series regression analysis of average monthly larval densities in Vila Assis Brasil before (September 2017–October 2018), during (November 2018–October 2019), and shortly after larviciding (November 2019). The solid line shows the trend based on least-squares linear regression fitted to empirical data (black dots) adjusted for a time-dependent variable (rainfall estimates 15 days prior to larval sampling) and for autocorrelation of residuals over time. The dashed vertical line indicates the time of the first larvicide application. No larvicide treatment was applied in May 2019 (see the main text for details)

The average larval density during the 14 months of pre-intervention monitoring of fish farming ponds, from September 2017 through October 2018, was 0.467 (95% CI, 0.444–0.490) anopheline larvae per dip. Larval density decreased to an average of 0.046 (95% CI, 0.041–0.051) larvae per dip from November 2018 through November 2019, following larvicide treatment from November 2018 through April 2019 and from June 2019 through October 2019 (Fig. 2). We fitted regression lines to pre- and post-intervention larval density data adjusted for rainfall 15 days prior to larval sampling (Fig. 3). The ITSA approach estimated the starting level of larval density at 0.690 larvae per dip, with a significant monthly decrease prior to the intervention by 0.033 (95% CI, 0.007–0.059, *P* = 0.016) larvae per dip, while adjusting for monthly rainfall. There was a further drop in larval density by 0.231 (95% CI, 0.010–0.452, *P* = 0.042) larvae per dip shortly after the intervention (November 2018), which characterizes a change in level [26], with sustained low larval densities until November 2019 (Fig. 3). No treatment was carried out in May 2019, leading to a transient rise in larval density (Fig. 2), but ITSA results remain unchanged if this sampling point was omitted (data not shown).

### Malaria prevalence before and during larvicide application

**Fig. 4:**
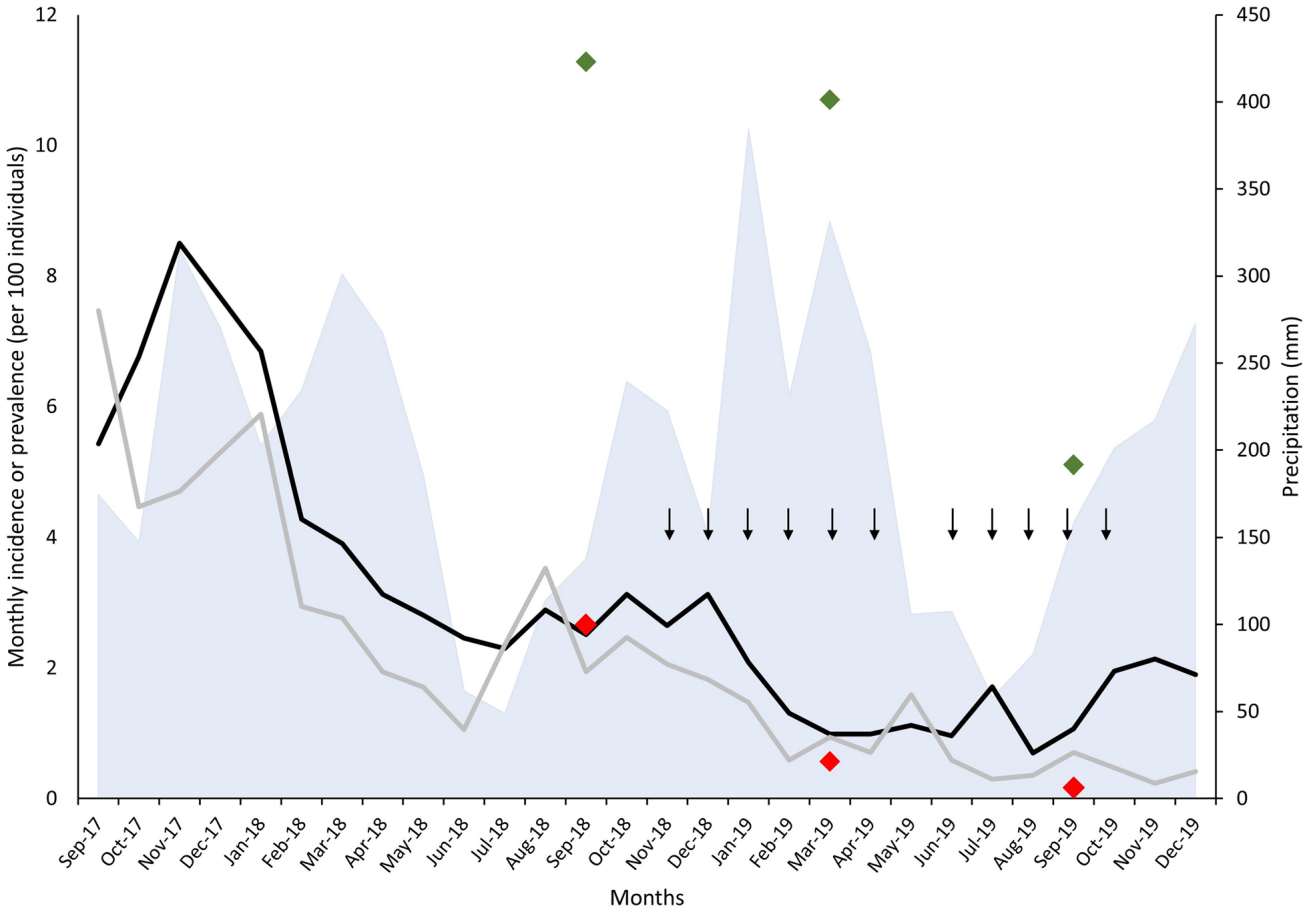
Malaria incidence and prevalence in Vila Assis Brasil (intervention site) and nearby larvicide-untreated localities in northwestern Brazil. The monthly incidence of microscopy-confirmed clinical malaria (*P. vivax* and *P. falciparum* infections combined) in Vila Assis Brasil (VAB) and comparison localities between September 2017 and December 2019 is shown as gray (VAB) and black lines (seven comparison localities in the municipality of Cruzeiro do Sul). Prevalence rates measured by microscopy and PCR in VAB before (September 2018) and during (March 2019 and September 2019) the intervention are indicated by red and green diamonds, respectively. Vertical arrows indicate the timing of larvicide treatment, and the light blue area chart indicates the monthly rainfall (mm)

During the pre-intervention prevalence survey in September–October 2018, 1500 permanent residents in VAB were examined by microscopy. Of them, 40 (2.67%; 95% CI, 1.94–3.65%) had malaria parasites detected (red diamond in Fig. 4). There were 33 infections with *Plasmodium vivax*, six with *P. falciparum,* and one mixed-species infection with *P. vivax* and *P. falciparum.* Prevalence rates dropped substantially in the following surveys. In March 2019, only eight of 1416 individuals examined (0.56%; 95% CI, 0.26–1.15%) had malaria parasites detected by microscopy (4.8-fold decrease in prevalence), with seven instances of *P. vivax* infection and only one *P. falciparum* infection. Finally, 1185 individuals were examined in September–October 2019, and only two (0.17%, 95% CI, 0.03–0.68%) were positive, both for *P. vivax* (15.6-fold decrease in prevalence compared with the baseline).

The proportions of positive genus-specific PCR results per survey are shown as green diamonds in Fig. 4. Of 1498 individuals tested in September–October 2018, 169 (11.28%; 95% CI, 9.72–12.99%) were positive. The proportion of PCR-positive individuals remained nearly unchanged in March 2019: of 1374 individuals tested, 147 (10.69%; 95% CI; 9.11–12.45%) were positive. However, we found a more substantial decline in PCR positivity in September–October 2019. At the end of the intervention, 1076 individuals were tested for malaria parasites by PCR, and 55 (5.11%; 95% CI, 3.87–6.60%) were positive (2.2-fold decrease in prevalence compared with the baseline). The proportion of malarial infections that were subpatent (i.e., missed by microscopy but detected by PCR) increased substantially during and shortly after the intervention, compared with the baseline survey. Accordingly, PCR detected 4.2-fold more infections than microscopy in the first survey, 19.1-fold in the second survey, and 30.1-fold more infections in the third survey.

### Malaria incidence before and during larvicide application

**Fig. 5:**
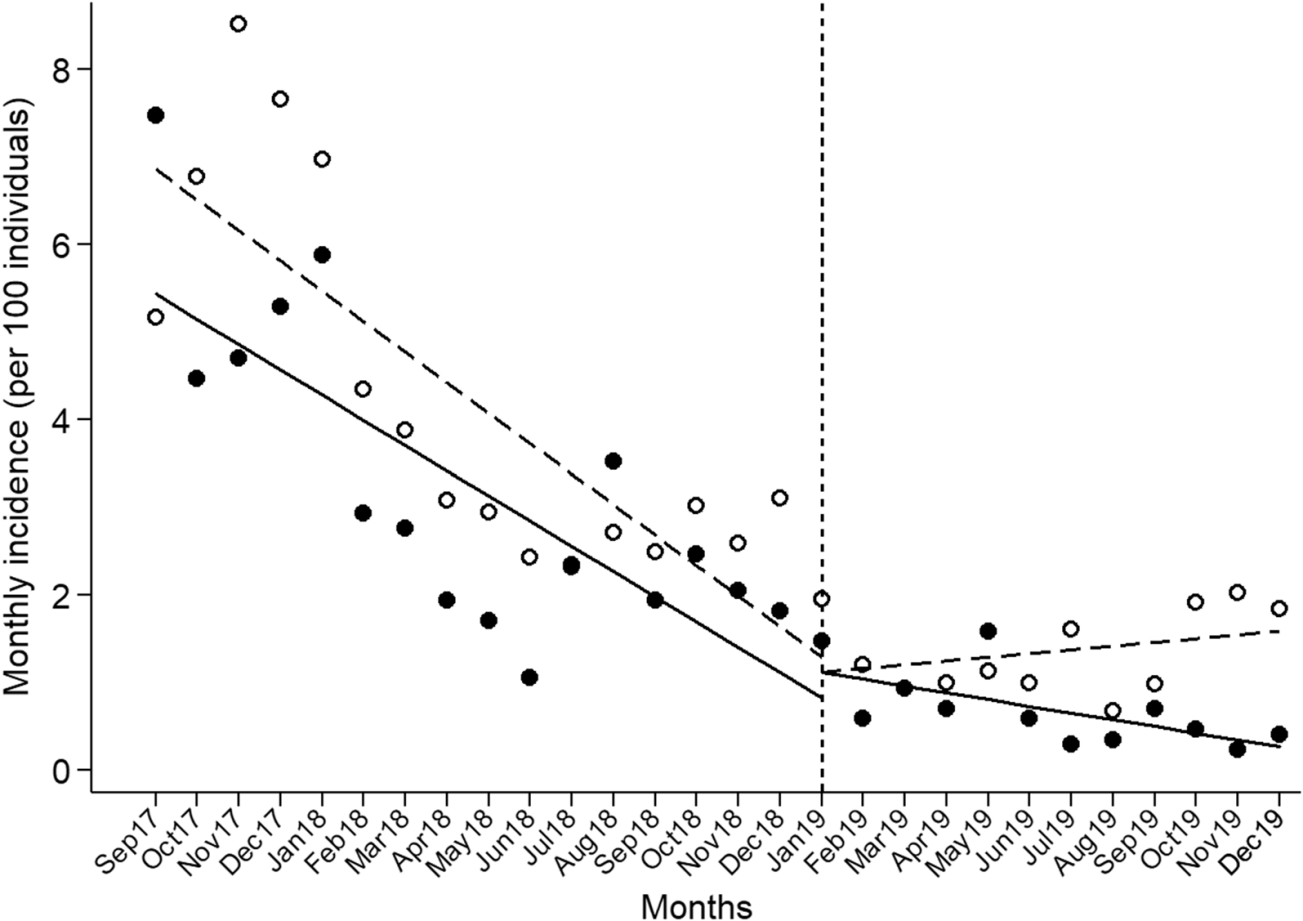
Interrupted time-series regression analysis of malaria incidence in Vila Assis Brasil (black dots and solid lines) and comparison localities (open dots and dashed lines) before and after the larviciding intervention. The least-squares linear regression model fitted to empirical data (dots) is adjusted for autocorrelation of residuals over time. The vertical dashed line separates pre- and post-intervention incidence measurements. The intervention started in November 2018, but we include only incidence estimates obtained from January 2019 in the “post-intervention period,” as we expect a 2-month delay in the effect of larval density reduction on malaria transmission (see “Methods”)

We compared the monthly incidence of malaria in VAB and comparison localities before and during the intervention using routinely collected morbidity data (Fig. 4). As expected, given the selection criteria applied, malaria rates dropped at similar rates in VAB and the comparison group from September 2017 through December 2018, with average incidence rates of 3.27 cases per 100 person-months (95% CI, 1.06–7.47) in VAB and 4.27 cases per 100 person-months (95% CI, 2.30–8.50) in the seven comparison localities altogether. Between January 2019 (2 months after the first larvicide application) and December 2019 (2 months after the last larvicide application), malaria incidence continued to decline in VAB, but not in the comparison localities (Fig. 4). The average incidence for the 12-month intervention period was 0.70 cases per 100 person-months (95% CI, 0.23–1.59) in VAB and 1.41 cases per 100 person-months (95% CI, 0.69–2.14) in the comparison group. No drop or change in incidence level was observed shortly after introducing the intervention (Fig. 5). Using ITSA, we estimate that malaria incidence declined by 0.08 (95% CI, 0.04–0.11) malaria cases per 100 person-months (*P* < 0.0001) between January and December 2019 in VAB, but remained nearly unchanged in comparison localities (nonsignificant increase by 0.04 malaria case per 100 person-months, *P* = 0.3031) (Fig. 5). The average difference in malaria incidence was 0.12 (95% CI, 0.03–0.21) cases per 100 person-months in VAB relative to the comparison group (*P* = 0.0080). This difference translates to 24.5 (95% CI, 6.2–42.8) microscopy-positive clinical malaria cases averted between January and December 2019 among the 1700 VAB residents.

## Discussion

Larviciding aims to reduce the population size of malaria vectors by killing the aquatic immature forms, so that fewer will develop into adults and transmit the parasite. Surprisingly few studies have examined whether chemical or biological larviciding can reduce malaria transmission across endemic settings with different primary vectors [27]. We have previously shown that 20 kg/ha of VectoMax FG has a substantial residual effect against *An. darlingi* larvae and pupae, lasting for at least 4 weeks, when applied to natural or human-made fish ponds [19]. Here, we investigated, for the first time, the impact of biological larviciding on community-wide malaria incidence in the Amazon. We show a tenfold reduction in average larval densities in treated fish farming ponds, compared to pretreatment averages, with a modest but significant decrease in malaria incidence rate associated with monthly VectoMax FG application, compared with non-intervention localities.

Our findings have major public health implications for malaria control and elimination across the Amazon. Malaria transmission associated with commercial aquaculture is increasingly common in the region [10, 11, 28, 29]. Indeed, in the mid-2000s, shortly after the Acre State government launched the Program for the Development of Fish Farming in the Juruá Valley, Brazil, explosive malaria outbreaks were recorded across the region [11, 29]. Fish farming ponds within and near urban centers pose a major risk of malaria transmission in densely populated areas in the Amazon [7, 11, 29]. Importantly, larviciding may be more effective to suppress outdoor biting and resting mosquitoes, such as the primary Amazonian malaria vector *An. darlingi* [2, 17], than core vector control interventions that target endophilic and endophagic anophelines, such as LLIN distribution and IRS [30].

Cluster-randomized controlled trials (cRCTs) are ideally suited for the evaluation of community-wide interventions. However, only a single published cRDT, carried out in Sri Lanka nearly 20 years ago, has evaluated the effectiveness of (chemical) larviciding for malaria control [31]. This cRDT, included in a recent systematic Cochrane review [27], found a nearly fivefold decrease in malaria incidence in intervention villages (5 cases per 100 person-years) compared with control villages (23 cases per 100 person-years). Larviciding is rarely assessed using cRCTs, in part because of the high cost and the complex logistics of these studies, with substantial risk of intervention spill-over affecting nearby control communities [30]. Conversely, ITSA with comparison group(s) has been increasingly used to measure the effectiveness of community interventions [32, 33] and may provide a cost-effective alternative to cRCTs in the evaluation of larviciding and other malaria control interventions.

Our study has some limitations. First, in contrast with cRDTs, we did not have a true control arm. Of note, malaria incidence rates have decreased in both VAB and the comparison localities during the 14-month pre-intervention period, but the rate of decline changed during the intervention (Fig. 4), highlighting the critical need for an appropriate comparison group in our ITSA approach. Second, we may have underestimated malaria incidence rates in VAB and comparison localities. We retrieved malaria morbidity data from an electronic case notification database that is assumed to comprise nearly all clinical malaria episodes diagnosed by microscopy and treated in CZS [23], but transient or chronic submicroscopic parasitemias that do not develop into clinically apparent infections detectable by conventional microscopy remain mostly undetected. Importantly, the proportion of malarial infections that were missed by microscopy but diagnosed by a more sensitive molecular method increased substantially during the intervention. Third, we measured malaria prevalence rates by using microscopy and molecular diagnostic methods in VAB but not in the comparison localities. Therefore, we cannot determine to what extent the sharp decline in the prevalence of patent infections in consecutive cross-sectional surveys in VAB, with a much less marked change in the prevalence of subpatent infections, results from the intervention or merely reflects an overall trend in malaria transmission in the region. Finally, we have not systematically characterized the anopheline fauna in VAB and comparison localities, where fish farming ponds are much less abundant, but available data show a significant variation in species abundance and composition in the anopheline communities from localities separated by 10–30 km across Juruá Valley [18].

In conclusion, our results suggest that the periodic application of biological larvicides with extended activity represents a valuable complementary malaria vector control strategy in commercial aquaculture settings of the upper Juruá Valley, northwestern Brazil. Further studies are warranted to examine the generalizability of these findings across the Amazon, especially in areas where larval habitats are present in widely different sizes and anopheline species other than *An. darlingi* contribute significantly to malaria transmission.

## Data Availability

Data supporting the conclusions of this article are included within the article. The data sets used are available at the University of São Paulo data repository (https://uspdigital.usp.br/repositorio/). Researchers who are interested in potential collaboration should contact the corresponding author (muferrei@usp.br).

## Abbreviations

CI: Confidence interval
cRCT: Cluster-randomized controlled trial
CZS: Cruzeiro do Sul
IRS: Indoor residual spraying
ITSA: Interrupted time-series analysis
LLIN: Long-lasting insecticidal net
VAB: Vila Assis Brasil
